# Enhanced identification of endocrine disruptors through integration of science-based regulatory practices and innovative methodologies: The MERLON Project

**DOI:** 10.12688/openreseurope.17319.1

**Published:** 2024-04-12

**Authors:** Terje Svingen, Anna-Maria Andersson, Julianna Angelova, Marta Axelstad, Julie Bakker, Lisa Baumann, Anna Beronius, Nora Bouftas, Frederic Chalmel, Sofie Christiansen, Charlotte Cornil, Pauliina Damdimopoulou, Deepika Deepika, Martijn E. T. Dollé, Monica Kam Draskau, Margit Bistrup Fischer, Casper P. Hagen, Ellen Hessel, Marie Louise Holmer, Samantha Hughes, Genon Jensen, Hanna Katarina Lilith Johansson, Anders Juul, Vikas Kumar, Saurav Kumar, Aurélie Lardenois, Katharina M. Main, Severine Mazaud-Guittot, S. Jannicke Moe, Gylli Mola, Anne-Simone Parent, Rafael Pineda, Antoine Rolland, Anna Kjerstine Rosenmai, You Song, Antonio Suglia, Manuel Tena-Sempere, Lydia Wehrli, Johanna Zilliacus, Majorie van Duursen

**Affiliations:** 1 National Food Institute, Technical University of Denmark, Kgs. Lyngby, Region Hovedstaden, 2800, Denmark; 2 Department of Growth and Reproduction, Copenhagen University Hospital (Rigshospitalet), Copenhagen, Denmark; 3 International Center for Research and Training in Endocrine Disruption of Male Reproduction and Child Health (EDMaRC), Copenhagen University Hospital (Rigshospitalet), Copenhagen, Denmark; 4 Health and Environment Alliance (HEAL), Brussels, Belgium; 5 Neuroendocrinology Laboratory, GIGA-Neurosciences, University of Liege, Liege, Belgium; 6 Environmental Health and Toxicology, Amsterdam Institute for Life and Environment, Vrije Universiteit Amsterdam, Amsterdam, North Holland, 2800, The Netherlands; 7 Institute of Environmental Medicine, Karolinska Institutet, Stockholm, 17177, Sweden; 8 Univ Rennes, Inserm, Irset (Institut de recherche en santé, environnement et travail), Rennes, F-25000, France; 9 Department of Clinical Science, Intervention and Technology, Karolinska Institutet, Huddinge, 14186, Sweden; 10 Department of Gynecology and Reproductive Medicine, Karolinska Institutet, Huddinge, 14186, Sweden; 11 IISPV, Departament d' Enginyeria Quimica, Universitat Rovira i Virgili, Tarragona, Catalonia, 43007, Spain; 12 Centre for Health Protection, National Institute for Public Health and the Environment (RIVM), Bilthoven, The Netherlands; 13 Department of Clinical Medicine, Copenhagen University Hospital (Rigshospitalet), Copenhagen, Denmark; 14 Norwegian Institute for Water Research (NIVA), Oslo, 0579, Norway; 15 Department of Cell Biology, Physiology and Immunology / IMIBIC., University of Cordoba, Cordoba, 14012, Spain

**Keywords:** endocrine disruption, regulatory toxicology, adverse outcome pathways, sexual development, reproduction, gender, new approach methodologies, systems biology

## Abstract

The prevalence of hormone-related health issues caused by exposure to endocrine disrupting chemicals (EDCs) is a significant, and increasing, societal challenge. Declining fertility rates together with rising incidence rates of reproductive disorders and other endocrine-related diseases underscores the urgency in taking more action. Addressing the growing threat of EDCs in our environment demands robust and reliable test methods to assess a broad variety of endpoints relevant for endocrine disruption. EDCs also require effective regulatory frameworks, especially as the current move towards greater reliance on non-animal methods in chemical testing puts to test the current paradigm for EDC identification, which requires that an adverse effect is observed in an intact organism. Although great advances have been made in the field of predictive toxicology, disruption to the endocrine system and subsequent adverse health effects may prove particularly difficult to predict without traditional animal models. The MERLON project seeks to expedite progress by integrating multispecies molecular research, new approach methodologies (NAMs), human clinical epidemiology, and systems biology to furnish mechanistic insights and explore ways forward for NAM-based identification of EDCs. The focus is on sexual development and function, from foetal sex differentiation of the reproductive system through mini-puberty and puberty to sexual maturity. The project aims are geared towards closing existing knowledge gaps in understanding the effects of EDCs on human health to ultimately support effective regulation of EDCs in the European Union and beyond.

## List of Abbreviations

ADME: absorption, distribution, metabolism, and excretion

AO: Adverse Outcome

AOP: Adverse Outcome Pathway

AOPN: Adverse Outcome Pathway Network

CLP: Classification, Labelling and Packaging

DHT: Dihydrotestosterone

EDC: endocrine disrupting chemical

EAS: oestrogen, androgen, and steroidogenesis

EU: European Union

IATA: Integrated Approaches to Testing and Assessment

KE: Key Event

KER: Key Event Relationship

NAM: New Approach Methodology

OECD: Organisation for Economic Co-operation and Development

PBTK: Physiologically Based Toxicokinetic

qAOP: quantitative Adverse Outcome Pathway

WHO: World Health Organization

## Introduction

Endocrine disrupting chemicals (EDCs) are of great concern to human and environmental health. The European Union (EU) considers the identification and regulation of EDCs a high priority and much effort is being put towards improving test methods and regulatory frameworks. Despite these efforts, many challenges remain before we have a system that is robust, reliable, and effective enough to adequately capture and regulate EDCs that may pose a threat to human health. These challenges include i) the lack of a sufficient number of validated test methods that can detect all relevant effects of EDCs, ii) incomplete knowledge about the capacity of many alternative test methods to predict effect outcomes in humans and laboratory animals, iii) interspecies variations or iv) sex differences within same species, and v) incomplete quantitative understanding of causal pathways of endocrine disruption. In addition, the regulation of EDCs within the EU remains tediously slow, as it typically involves substance evaluation on a one-by-one basis with a demand for extensive animal studies. With 26.000 substances currently registered under REACH ( ECHA, 2024) and estimated 40-60.000 substances in global commerce ( WHO, 2022), there is an urgent need for speeding up the process.

Sexual development is especially sensitive to EDCs due to its intrinsic dependence on hormone signalling at critical life stages. During specific time periods, such as foetal and early childhood, exposure to EDCs can disrupt the normal signalling pathways, potentially leading to long-term adverse effects on the reproductive system ( Johansson *et al.*, 2017; Lopez-Rodriguez *et al.*, 2021; Sharpe, 2020; Skakkebaek *et al.*, 2016). These early-life exposures are of great concern since they can induce irreversible effects that potentially impact reproductive organs, fertility, and the overall functioning of the reproductive system throughout life.

Our knowledge about normal mammalian sexual development and later reproductive function is substantial and includes detailed characterization of numerous molecular mechanisms and cellular events. Several established and validated test methods for evaluating endocrine disruption are also available, with some related to developmental and reproductive toxicity testing (OECD GD 150) ( OECD, 2018), which goes some way of informing on the potential effects of EDCs on both male and female reproduction. However, the current catalogue of test methods is not sufficient, and it remains challenging to predict, with reasonable accuracy, many adverse health outcomes in animal models or humans based on data from alternative test methods alone ( Svingen *et al.*, 2022). The MERLON consortium, funded under EU’s Horizon Health programme, aims to address these challenges, and provide a roadmap to advance EDC identification within the EU.

In this article we outline the concept and rationale for the MERLON project. First, we provide a short overview of how EDCs can affect the reproductive system and cause a variety of adverse effects depending on what life stages disruption occurs. This is followed by an emphasis on the regulatory needs for EDC identification, before outlining the MERLON project’s objectives and research strategies.

## EDC exposure and effects on reproductive development and function – a short overview

EDCs are substances that can interfere with the normal functioning of the endocrine system in humans and wildlife and thereby cause adverse effects. They can act by numerous mechanisms, such as mimicking, blocking, or disrupting the production, transport, release, metabolism, or action of endogenous hormones and cause a plethora of adverse effects in living organisms. Adverse effects include developmental toxicity, reproductive disorders, and disrupted hormone-dependent physiology. EDCs are now ubiquitous in the environment and are found in everyday products such as pesticides, plastics, cosmetics, food packaging materials, paints *etc*, and their potential impact on human health continues to raise significant concern ( EC, 2012; EEA, 2019).

**Figure 1.**
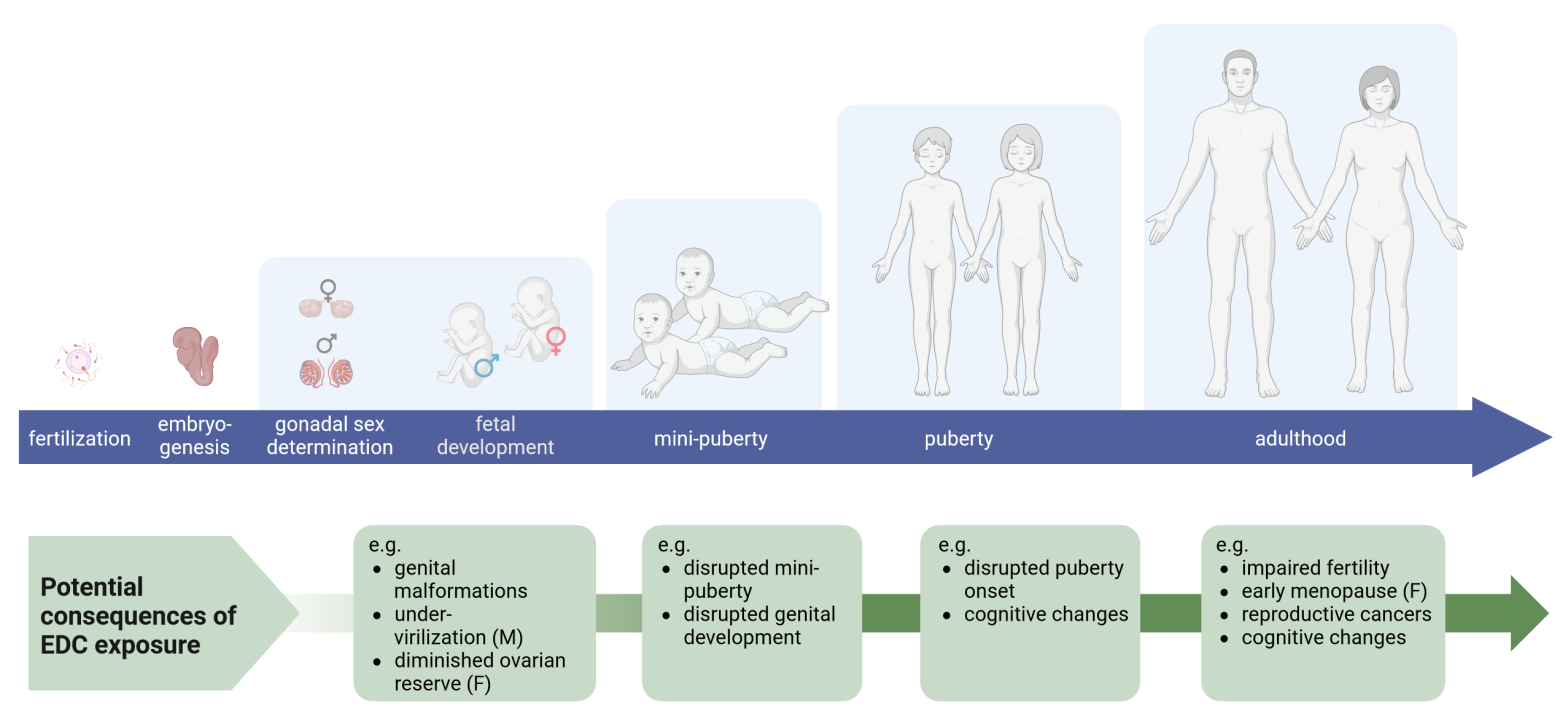
Human life stages for endocrine disruption and disease manifestations. Sexual development initiates at fertilization with chromosomal sex determination, but phenotypic bifurcation of the biological sexes is first visible immediately after gonadal sex determination; differentiation of either testes or ovaries in XY and XX foetuses, respectively. Subsequent sexual development hinges on steroid sex hormones, not least high androgen levels produced by the foetal testes in males. Since reproductive development is strongly influenced by relative hormone levels, reproductive development from foetal stages through mini-puberty and puberty are vulnerable to endocrine disrupting chemicals (EDCs). Life-long exposure, or exposures at various life stages, can lead to a range of reproductive disorders, from severe malformations to a shift in puberty onset and infertility. Created with BioRender.com.

Both sexual development and reproductive function are dependent on hormone signalling, and thus vulnerable to EDCs. Exposures have been linked to a range of reproductive disorders in both sexes, with effects manifesting at birth or later in adulthood ( Figure 1). This has been repeatedly shown in both animal models and human epidemiological studies ( Johansson *et al.*, 2017; Land *et al.*, 2022; Palanza *et al.*, 2021; Skakkebaek *et al.*, 2016). Importantly, critical windows of exposure are paramount when delineating effects on the reproductive systems, since hormone signalling pathways have unique functions that differ throughout life. In general terms, one may consider hormone action during early development as instructions for building the reproductive system whereas during adulthood hormones make the reproductive system function. As such, endocrine disruption during foetal stages and early childhood are especially concerning as the adverse effects may be irreversible.

In males, developmental exposure to EDCs has been linked to disorders such as cryptorchidism, hypospadias, reduced fertility, and reproductive cancers. Disorders arising in consequence of malfunctioning testes during foetal life have been described under the Testicular Dysgenesis Syndrome hypothesis ( Skakkebaek *et al.*, 2001). However, male reproductive disorders triggered by exposure to EDCs may also arise from other causes ( Svingen *et al.*, 2022). Nevertheless, since male sexual differentiation is largely controlled by androgen signalling during foetal life, primarily by testosterone and dihydrotestosterone (DHT), males are particularly sensitive to endocrine disruption during this life stage. A surge in androgen production is observed two-thirds into gestation in mice and rats (gestational day ~16–21) and at the start of second trimester in humans (gestational weeks ~12–20), commonly referred to as the masculinization programming window ( Welsh *et al.*, 2008). This androgen surge is not occurring in females, as they do not have testes, and hence the difference in androgen levels is the main driver of sexually dimorphic differentiation of the sexual phenotypes following gonadal differentiation into either testes or ovaries. This is also why many of the EDCs causing male reproductive disorders have either estrogenic or anti-androgenic potentials, which may lead to under-masculinization seen with for instance shorter anogenital distance ( Schwartz *et al.*, 2019), hypospadias ( Mattiske & Pask, 2021), and cryptorchidism ( Bay & Anand-Ivell, 2014) as well as increased risk of testicular cancer and reduced semen quality in adulthood ( Skakkebaek *et al.*, 2001).

In females, developmental exposure to EDCs have been linked to disorders such as precocious puberty, reduced fertility, early menopause, and reproductive cancers ( Johansson *et al.*, 2017). Importantly, the pool of ovarian primordial follicles is established during early foetal life and is deciding for a woman’s reproductive lifespan. During folliculogenesis, primordial follicles are formed by granulosa cells surrounding germ cells as they enter and arrest in the first meiotic division. The transition from mitosis to meiosis is highly regulated, and if the process is disrupted, germ cells are lost to apoptosis ( Jung & Kee, 2015). Studies suggest that the germ cell lineage, and follicles, are particularly vulnerable to EDCs and may have latent effects on adult fertility. Reduction of the follicle pool may also affect age at pubertal onset and cause premature menopause ( Dean *et al.*, 2016).

Mini-puberty is a transient activation of the hypothalamic-pituitary-gonadal axis during infancy resulting in adult levels of androgens in boys as well as maturation of ovarian follicles and oestradiol production in girls ( Busch *et al.*, 2022; Kuiri-Hänninen *et al.*, 2011; Ljubicic *et al.*, 2022a). This transient period of hormonal activity is thought to play a role in the development of the reproductive system and has been associated with certain physiological changes, including the growth of reproductive organs and the maturation of reproductive tissues ( Boas *et al.*, 2006; Ljubicic *et al.*, 2022b; Main *et al.*, 2000). Mini-puberty serves as a window of opportunity to evaluate, and in certain cases treat, gonadal dysfunction caused by disrupted foetal pituitary-gonadal axis development ( Kuiri-Hänninen *et al.*, 2014; Main *et al.*, 2000). The hormonal changes that take place during mini-puberty are considered essential for establishing adult reproductive function ( Scheutz Henriksen *et al.*, 2022) and disruption to this process by EDC exposure may thus have long-term negative consequences. There is evidence for EDCs disrupting mini-puberty, including phthalates ( Muerköster *et al.*, 2020) and parabens ( Jensen *et al.*, 2021).

Changes in puberty onset have been associated with EDC exposure in both sexes ( Lopez-Rodriguez *et al.*, 2021). These may arise from exposures during the time of pubertal development itself, but worryingly also by foetal and perinatal exposure. Puberty onset is controlled by hormonal release from the hypothalamic-pituitary axis, but also requires proper feedback from gonadal hormone signalling ( Argente *et al.*, 2023). Hence, both compromised brain and gonad development can lead to disrupted puberty, and it can be challenging to unravel where along the signalling axis disruption is initiated ( Uldbjerg *et al.*, 2022). There may also be sex-specific effects in human, suggested by phthalates having been associated with delayed puberty in girls but precocious puberty in boys ( Berger *et al.*, 2018).

Sex is genetically defined in mammals and typically defines individuals as being either male or female. Gender, on the other hand, can be considered a social construct in humans and refers to a personal intrinsic sense of self; it typically defines if individuals view themselves as man or woman, or other ( Coleman *et al.*, 2022; Swaab *et al.*, 2021). It has been demonstrated in animals and humans that steroid hormones influence brain development during perinatal life resulting in sex differences in brain structure, as well as differences in psychosexual characteristics in humans ( Bakker, 2022). In rodent toxicity studies, developmental exposure to different EDCs has been demonstrated to affect both reproductive and cognitive behaviour in offspring ( Ducroq *et al.*, 2023; Hilz & Gore, 2022). Since animals do not have a gender, it is more difficult to ascertain direct effects of EDCs on gender aspects in controlled experiments. Notably, however, there has been a significant increase in persons referred to health care clinics with gender incongruence over the last decades ( Hilden *et al.*, 2021; Indremo *et al.*, 2021; Kuyper & Wijsen, 2014; Van Caenegem *et al.*, 2015). Additional research seeking to find explanations for this steady rise in incidence rates is therefore warranted, including potential involvement of EDCs.

## Improving testing methodologies and EDC identification

In recent years, great efforts have been put into updating standardised test guidelines and developing new methods for detecting endocrine disrupting effects. Major advances have been made and criteria for identification of EDCs have been introduced into various regulations. Despite this, we still lack test methods to adequately detect several important effect endpoints and the regulatory system remains tediously slow; only a small number of substances are currently regulated based on inherent endocrine disrupting properties. One major challenge for EDCs has been the required evidence for an ‘adverse effects in intact organism’. This requirement is embedded in the definition of EDCs provided by the World Health Organization ( WHO/IPCS, 2002), and consequently in the scientific criteria for identifying EDCs under the EU regulations for plant protection products ( EU, 2018) and biocides ( EU, 2017), and recently also in the Classification, Labelling and Packaging (CLP) Regulation ( EU, 2023). The new CLP criteria also introduces the possibility to use non-animal data with an equivalent predictive capacity as human and animal data for EDC identification, which may pave the way for a wider application of new approach methodologies (NAMs: herein meaning *‘alternatives to traditional animal toxicity testing’*).

A 2019 report commissioned by the European Parliament’s Committee on Petitions estimates the annual cost related to the effects of exposure to EDCs at 163 billion Euros, with 5% (8.15 billion euros) attributed to reproductive disorders ( Kassotis *et al.*, 2020). Apart from the monetary costs to society, reproductive disorders such as infertility, genital malformation, or reproductive cancers can cause significant personal suffering. The societal need for robust and efficient testing regimens is therefore clear. Yet, for reproductive toxicity we still lack both appropriate methods and knowledge that is needed to predict human-relevant outcomes based on test data, being it *in silico*, *in vitro* or *in vivo*. 

To support public authorities with scientific evidence in safety assessment of EDCs, the MERLON project aims to provide a roadmap to effectively assess effects on sexual differentiation and function arising from endocrine disruption for substances under assessment in EU regulatory frameworks. A clear goal is to practically implement new and emerging concepts of 21 ^st^ Century toxicology, such as adverse outcome pathways (AOPs), and NAMs that are compliant with the 3R-principles to refine, reduce and replace animal testing. This will facilitate informed EDC identification by providing necessary mechanistic knowledge and ultimately support various regulatory frameworks, including biocidal products, plant protection products, REACH chemicals, cosmetics, and pharmaceuticals, as well as the CLP regulation.

The AOP framework is well suited for predictive toxicology as it provides empirically supported knowledge about causal pathways linking initial stressor events with adverse outcomes (AOs) in intact organisms ( Villeneuve *et al.*, 2014). By organizing biological, especially mechanistic, knowledge into modular units, including key events (KEs) that describe measurable effects in a biological system and KE relationships (KERs) that describe causal links between two KEs, predictive toxicological pathways can be constructed. In MERLON we will leverage the AOP concept and build on an emerging AOP network (AOPN) for reproductive toxicity to facilitate more integrated use of NAMs in EDC identification. Novel approaches will be applied for quantification of AOPs, including Bayesian network modelling ( Moe *et al.*, 2021) and piecewise structural equation modelling ( Cao *et al.*, 2023).

Due to the central role steroid hormones play in reproductive development and function, it is not surprising that many adverse effects induced by EDCs may occur through the estrogenic, androgenic, or steroidogenic (EAS) modalities ( ECHA/EFSA *et al.*, 2018; OECD, 2018). This background knowledge is also why most attention has been paid to these modalities over recent decades of EDC-induced reproductive toxicity and why the emerging AOPN for reproductive toxicity in the AOP-wiki includes several adverse outcomes (AOs) known to be mediated through EAS disruption ( Wiklund *et al.*, 2023; Zilliacus *et al.*, 2024). Notably, however, this does not exclude the involvement of other modalities such as, for example, retinoid signalling.

## The MERLON approach and methods

**Figure 2.**
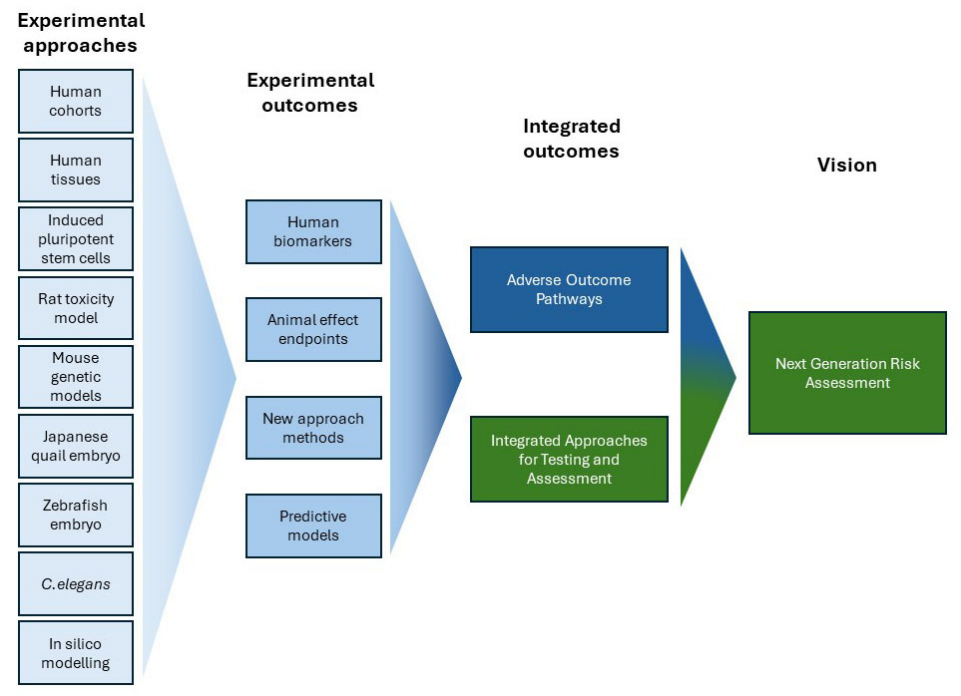
Applying basic research to improve next generation risk assessment of endocrine disrupting chemicals (EDCs). The MERLON project (blue boxes) will employ a broad array of research methods to establish a roadmap for the identification of EDCs with greater reliance on alternative (reductionist) methods and new approach methodologies (NAMs). Using human cohorts and foetal tissues together with rodent toxicity and genetic models, human-relevant biomarkers and assays will be pinpointed and subsequently exploited to establish alternative test methods, including zebrafish, nematode and quail models. In addition, modelling approaches will supplement empirical data in providing quantitative extrapolative frameworks for predicting adverse outcomes in humans based on non-animal test results. This will be achieved by continued improvement of an Adverse Outcome Pathway Network (AOPN) for reproductive toxicity that can be further leveraged in future development of integrated approaches for testing and assessment (IATA), ultimately supporting next generation risk assessment (NGRA) more broadly.

In addition to regulatory tests that are performed in compliance with internationally validated test guidelines, data from other sources such as non-standard research studies or non-validated methods can also provide relevant information for EDC assessments. In MERLON we will use and develop a broad range of experimental approaches chosen specifically to facilitate increased used of integrated approaches for EDC identification ( Figure 2). To achieve our aims, MERLON will address four main objectives:

i)     Provide human data on the role of EDC exposure during foetal development and changes in mini-puberty, puberty, reproductive function, and gender incongruence using existing biobanks and cohorts.

ii)     Develop NAMs focusing on sexual development and function and the effects of EDCs thereon.

iii)     Develop a roadmap for EDC identification, making the best use of NAMs.

iv)     Consult, engage, and collaborate with relevant stakeholders, including citizens and public authorities such as EU risk assessment bodies and regulators.

### Objective 1: Biomarkers and association between EDC exposures and effects on developmental and reproductive toxicity in humans

The first objective involves utilizing ongoing human cohorts and biobanks to provide human data on the correlation between foetal exposure to EDCs and the perturbation of reproductive organ development, mini-puberty, and puberty. Further data on the potential influence of EDCs on sexually dimorphic neurodevelopment and their impact on gender identity will also be provided. Leveraging human cohorts and tissues will enable the identification of predictive biomarkers for future epidemiological studies, as well as lending support to AOP development and use. Additional focus will be on elucidating health endpoints that currently lack sufficient data, such as disrupted mini-puberty and gender incongruence. The project aims to advance our capabilities of identifying EDCs by providing improved biomarkers for predicting health outcomes. A key goal is to pinpoint the most sensitive windows of susceptibility to EDC exposure in humans during development and provide important information for refining our understanding of the risks associated with these chemicals throughout different life stages.

### Objective 2: New approach methodologies for EDC identification

The second objective involves the development of NAMs for EDC identification, including using non-mammalian, whole-animal models such as non-protected life stages of zebrafish (<120 hpf embryo), nematodes, and Japanese quail. Traditional and genetically modified rodent models will be used to gain mechanistic knowledge, including in-depth interrogation of novel modes of action. We will employ both bulk- and single-cell transcriptomics approaches to gain mechanistic information on the effects of EDC exposures on various organs and aid in cross-species comparisons of EDC-mediated effects. Human induced pluripotent stem cells will be used to gauge how hormones such as androgens and oestrogens contribute to sex differences in brain development. Zebrafish embryo models will be used to study EDC-induced (neuro) developmental toxicity and early markers of sexual development from which the EDC-induced effects could potentially be predicted. These early markers may subsequently be implemented in existing zebrafish embryo-assay to assess the effects of selected EDCs on biomarkers of sexual differentiation and on the developing brain. *C. elegans* models will be used to assess multi-generational inherited effects from EDC exposure. As with the human studies, special emphasis will be placed on exposure during critical life stages, identification of new biomarkers and on exploring endpoints that are currently not adequately addressed in internationally accepted test guidelines. New mechanistic knowledge and empirical evidence will be used to support and expand the abovementioned AOPN. The use of Physiologically Based Toxicokinetic (PBTK) models will address multi-organ exposures and physiological barriers, enhancing the applicability of NAMs for EDC identification. In addition, cross-scale (e.g., *in vitro* to *in vivo*) dose extrapolation by PBTK modelling will allow for better utilization of the NAMs data to support the development of quantitative AOP (qAOP) models. Ultimately, these predictive approaches will allow more reliable estimations of thresholds (points of departure) necessary to induce adverse effects and support the use of NAMs in regulatory decision-making.

### Objective 3: A roadmap to advance EDC identification in EU regulations

The third objective involves conducting case studies to evaluate the application of an AOPN on EAS-mediated reproductive effects, drawing additional insights from Objectives 1 and 2. We will assess the utility of AOPs and qAOPs in EDC assessment and identification, and assess the capacity of AOPNs to capture intricate interactions across biological levels of complexity. Additionally, of absorption, distribution, metabolism, and excretion (ADME) parameters will be incorporated in prediction models. In this way, we will develop a roadmap for EDC identification, emphasizing the optimal use of NAMs, focusing on the predictability and associated uncertainties in EDC identification. With increasing emphasis on mechanism-based NAMs approaches, the project aims to provide recommendations on methods, methodologies, and principles. This includes leveraging NAM data, implementing strategies for grouping chemicals, read-across, and conducting cross-species extrapolations to enhance the identification of EDCs.

### Objective 4: Consult, engage and collaborate with relevant stakeholders

The fourth objective involves engagement with citizens and key public authorities, such as EU risk assessment bodies and regulators, with the goal to develop fit-for-purpose framework enhancements that align with current practices for EDC identification within the EU. The MERLON consortium, along with its Scientific Advisory Board, maintains robust connections with regulatory bodies, societal entities, and scientific networks, including the Partnerships for the Assessment of Risks from Chemicals (PARC, https://www.eu-parc.eu/). Ongoing collaboration with the Joint Research Centre (JRC) and the OECD Test Guidelines Program (TGP) will ensure uniform integration of the project outcomes with global initiatives related to EDC testing and identification. Finally, considerations for gender, regional variations, socioeconomics, and cultural factors are integral to our approach, as we seek to enhance the societal impact of our research activities. Collaborations with other projects of this call, the ENKORE cluster, will optimize synergies and amplify the overall impact of MERLON.

## Concluding remarks

Although many mechanistic endpoints related to reproductive toxicity can be assessed in rodent studies, the existing list of validated NAMs methods to test for effects mediated by the EAS modalities is notably deficient. The critical knowledge gap lies in the quantitative relationships between early signs of perturbation and adverse effect outcomes of regulatory concern. The MERLON project strategically addresses this challenge by focusing on the development and application of a AOPN for reproductive toxicity. This approach, incorporating both quantitative and qualitative inputs, is envisioned to significantly enhance the predictive capacity of NAM data, contributing to the development of fit-for-purpose NAMs that can either replace or complement *in vivo* toxicity studies alongside human epidemiological data.

## Ethics and consent

Ethical approval and consent were not required for writing this summary paper. All planned experiments under MERLON are covered by valid ethical licenses.

## Disclaimer

The views expressed in this article are those of the authors. Publication in Open Research Europe does not imply endorsement of the European Commission.

## Data Availability

No data are associated with this article.
