# Supplementary material for: Enhanced identification of endocrine disruptors through integration of science-based regulatory practices and innovative methodologies: The MERLON Project
Source: Open Res Eur. 2024 Apr 12;4:68. [Version 1] doi: 10.12688/openreseurope.17319.1 (PMC11179054; doi:10.12688/openreseurope.17319.1)
Supplement: Peer review history

# Reviewer report: Reviewer response for version 1

**DOI:** 10.21956/openreseurope.18719.r40908

**Published:** 2024-06-14

**Authors:** Qi Wang

**Affiliations:** 1 School of Public Health, Peking University, Beijing, Beijing, China

This article provides a concise and insightful introduction to the MERLON Project, a highly promising initiative aimed at developing a comprehensive roadmap for identifying endocrine-disrupting chemicals (EDCs) using new approach methodologies (NAMs) within the European Union. The article is well-written, offering a thorough overview of the project's background, objectives, and methods.

 The MERLON Project addresses mechanistic insights and explores forward-looking strategies for NAM-based identification of EDCs, particularly through the development and application of the Adverse Outcome Pathway (AOP) network. The AOP framework is sound, and applying the AOP network to identify EDCs presents a significant challenge. However, there are uncertainties and limitations in predicting potential EDCs. It is essential to conduct case studies on both known EDCs and new chemicals, as the predictability for new chemicals may be limited due to insufficient test data.

 I suggest that the authors include the project's timeline to provide a clearer context for its progress. We eagerly anticipate the research outcomes of the MERLON Project.

Where applicable, are recommendations and next steps explained clearly for others to follow? (Please consider whether others in the research community would be able to implement guidelines or recommendations and/or constructively engage in the debate)

Not applicable

Does the article adequately reference differing views and opinions?

Partly

Are all factual statements correct, and are statements and arguments made adequately supported by citations?

Yes

Is the rationale for the Open Letter provided in sufficient detail? (Please consider whether existing challenges in the field are outlined clearly and whether the purpose of the letter is explained)

Yes

Is the Open Letter written in accessible language? (Please consider whether all subject-specific terms, concepts and abbreviations are explained)

Yes

Reviewer Expertise:

Computational Toxicology；PBPK modeling and risk assessment

I confirm that I have read this submission and believe that I have an appropriate level of expertise to confirm that it is of an acceptable scientific standard.

# Reviewer report: Reviewer response for version 1

**DOI:** 10.21956/openreseurope.18719.r40271

**Published:** 2024-06-08

**Authors:** Andrea Terron

**Affiliations:** 1 EFSA European Food Safety Authority, Parma, Italy

Please provide a full report, expanding on your answers to the questions above. In particular, if you answered “no” or “partly” to any of the questions, please give constructive and specific details as to how the authors can address any criticisms. Please indicate clearly which points must be addressed to make the article technically sound. ( *50 words min.*)

 The paper presents the status in the EU on how the assessment of the endocrine disrupting properties is conducted following the different jurisdictions for chemicals and clearly summarize the challenges and the gaps in the current approach. Based on these, is proposing a project mapped on criticalities and clearly stating the objectives and the main strategy to achieve them.

 The article reference to several stakeholders and is proportionate selection of adequate partner to seek collaboration with.

 The scientific considerations are correct, adequate, and well reflect the current uncertainties and areas of concerns for what is concerning the relationship between chemical exposure and endocrine disruption. The article is focusing on the mini puberty window of exposure which is recognized as a complex period for the impact of endocrine disrupting chemical and adequacy of animal models. The article is considering established scientific evidence as well circumstantial evidence and associated gaps.

 The article is easy to read and very clear.

 The article is not giving recommendations but as a scoping paper is framing the proposal on clear objectives and strategic plan. In this, the outcome of the project can well represent a step forward for the regulatory implementation of assays and the development of guidance or revisitation of existing guidance that needs to be updated to cover endpoints or window of exposure so far neglected.

Where applicable, are recommendations and next steps explained clearly for others to follow? (Please consider whether others in the research community would be able to implement guidelines or recommendations and/or constructively engage in the debate)

Yes

Does the article adequately reference differing views and opinions?

Yes

Are all factual statements correct, and are statements and arguments made adequately supported by citations?

Yes

Is the rationale for the Open Letter provided in sufficient detail? (Please consider whether existing challenges in the field are outlined clearly and whether the purpose of the letter is explained)

Yes

Is the Open Letter written in accessible language? (Please consider whether all subject-specific terms, concepts and abbreviations are explained)

Yes

Reviewer Expertise:

Expertise in toxicological pathology and toxicology. Risk assessment for endocrine disrupting chemicals and neurotoxicants. My area of expertise fit very well with the items discussed in the paper

I confirm that I have read this submission and believe that I have an appropriate level of expertise to confirm that it is of an acceptable scientific standard.
